# Research progress of neoantigen-based dendritic cell vaccines in pancreatic cancer

**DOI:** 10.3389/fimmu.2023.1104860

**Published:** 2023-01-25

**Authors:** Xin Zhang, Zheng Xu, Xiangpeng Dai, Xiaoling Zhang, Xueju Wang

**Affiliations:** ^1^ Department of Pathology, China-Japan Union Hospital, Jilin University, Changchun, Jilin, China; ^2^ Key Laboratory of Organ Regeneration and Transplantation of Ministry of Education, First Hospital of Jilin University, Changchun, China; ^3^ National-Local Joint Engineering Laboratory of Animal Models for Human Disease, First Hospital of Jilin University, Changchun, China

**Keywords:** dendritic cell, pancreatic cancer, mutation burden, immunotherapy, cancer vaccines, neoantigen

## Abstract

The mutation of the crucial genes such as tumor suppressors or oncogenes plays an important role in the initiation and development of tumors. The non-synonymous mutations in the tumor cell genome will produce non-autologous proteins (neoantigen) to activate the immune system by activating CD4+ and CD8+ T cells. Neoantigen-based peptide vaccines have exhibited exciting therapeutic effects in treating various cancers alone or in combination with other therapeutic strategies. Furthermore, antigen-loaded DC vaccines are more powerful in inducing stronger immune responses than vaccines generated by antigens and adjuvants. Therefore, neoantigen-based dendritic cell (DC) vaccines could achieve promising effects in combating some malignant tumors. In this review, we summarized and discussed the recent research progresses of the neoantigen, neoantigen-based vaccines, and DC-based vaccine in pancreatic cancers (PCs). The combination of the neoantigen and DC-based vaccine in PC was also highlighted. Therefore, our work will provide more detailed evidence and novel opinions to promote the development of a personalized neoantigen-based DC vaccine for PC.

## Introduction

Cancer can be caused by the alteration of the expression of genes controlling cell growth. The mutation of the crucial genes such as tumor suppressors or oncogenes plays an important role in the initiation and development of tumors. The loss of function mutations in tumor suppressor genes are frequently found in multiple cancers that will fail to produce the protein or the protein will not function properly. Moreover, mutation, gene amplification, and chromosome rearrangements are the three main genetic mechanisms to activate oncogenes. The activation mutations of proto-oncogenes will cause structural changes in their encoded proteins and lead to uncontrolled, continuous activation of the oncoproteins. The loss of the function mutations of tumor suppressors and the activation mutations of oncogenes will cause uncontrolled cell growth and ultimately contribute to tumorigenesis. For example, Hui Cai et al. analyzed the mutational landscape of 153 gastric cancer patients by targeted next-generation sequencing and identified 35 significantly mutated genes, and the tumor suppressor *TP53* was found to be the most frequently mutated gene (1). Moreover, Kirsten rat sarcoma viral oncogene homolog (KRAS) mutation is one of the most common gene mutations and is a frequent driver in lung cancer, colorectal cancer, and pancreatic cancer (PC). KRAS mutations drive 85%–90% of PC cases, and the high prevalence of the oncogenic mutation of the KRAS gene is the hallmark of PC that plays a crucial role in the initiation and development of PC (2).

Importantly, non-synonymous mutations of the genes in the tumor cell genome will produce non-autologous proteins that can only be found in tumor cells (3, 4). These proteins have the capability to activate the immune system and are now known as neoantigens, which own strong immunogenicity and does not express in normal cells. Neoantigens can activate CD4+ and CD8+ T cells to induce immune response, and they are current novel and important targets of cancer immunotherapy (5). Therapeutic vaccination is one of the cancer immunotherapies and could regulate immune pathways to induce or enhance the inadequate antitumor immune responses (6). Therefore, the successful development of tumor vaccines targeting neoantigens through nucleic acid, dendritic cell (DC)–based and synthetic long peptide (SLP) vaccines might benefit the currently used immunotherapeutic strategy. The safety and immunogenicity of the personalized neoantigen vaccine NEO-PV-01 in combination with PD-1 blockage was firstly investigated in the treatment of advanced melanoma, non-small cell lung cancer, or bladder cancer patients, and no adverse events of the regimen were found (7). DCs are bone marrow–derived, morphologically and functionally heterogeneous cells with the capability to activate primary immune responses by presenting the antigens to naïve CD4+ and CD8+ T cells (8). Therefore, given the critical function of DCs in modulating the innate and adaptive immune responses and their high sensitization to tumor-associated antigens (TAAs), DCs are considered crucial players for the development of immunotherapies and a major focus of cancer vaccine development (6). DC-based immunotherapies, especially the DC-based vaccination, were widely studied in recent years, and the clinical trials for different DC-based vaccinations have exhibited positive effects on the induction of antitumor responses and prolonged survival of patients with different types of tumors (6).

Given the high tumor specificity and immunogenicity of neoantigens, neoantigens were thought to be one of the ultimate targets for tumor immunotherapy. Neoantigen-based peptide vaccines have exhibited exciting therapeutic effects on the treatment of glioblastoma (9). It was reported that antigen-loaded DC vaccines are more powerful in inducing stronger immune responses than vaccines generated by antigens and adjuvants (10). Therefore, neoantigen-based DC vaccines could achieve promising effects in combating some malignant tumors (11). In this review, we summarized and discussed the recent research progresses of the neoantigens and DC-based vaccines and the potential roles of neoantigens in the generation of neoantigen-based DC vaccines in the treatment of PC.

## Neoantigens of pancreatic cancer

Pancreatic ductal adenocarcinoma (PDAC), a highly aggressive cancer type, accounts for 85% of PCs with a 5% of 5-year survival rate (12). Surgical resection is the only effective approach to prolong the survival time of PDAC patients if PDAC is diagnosed. However, the surgical resection is no longer beneficial for the PDAC patients with metastasis. Moreover, chemotherapy, radiotherapy, or immunotherapy could block the development of PDAC but the survival time of treated patients can only be extended to several months. Due to the poor early detection and the specific invasiveness of PDAC, further in-depth investigations for additional therapeutic strategies with high selectivity are need to eradicate PDAC (12).

It was well known that the mutations of oncogenes and tumor suppressors are critical factors for tumorigenesis including PC. The most frequently mutated genes of PDAC are *KRAS*, *CDKN2A*, *TP53*, and SMAD family member 4 (SMAD4), which are also considered driver genes and correlated with the poor outcomes of resected PDAC patients (13). Furthermore, the other germline mutations in mismatch repair genes (MLH1, etc.), the partner and localizer of BRCA2 (PALB2), cationic trypsinogen-gene (PRSS1), serine/threonine kinase 11 (STK11), ATM serine/threonine kinase (ATM), and breast cancer 1 (BRCA1) and breast cancer 2 (BRCA2) are correlated with a high risk of PC (14–16). As early as 2008, the coding regions of more than 29,000 genes in pancreatic adenocarcinomas were sequenced and 63 genomic alterations were identified that covered a core set of 12 cellular signaling pathways. These pathways included KRAS signaling, cell cycle regulation, DNA damage, TGF-beta signaling, RNA processing, and WNT signaling (13, 17) (**Table 1**). Further analysis of the *KRAS* types indicated that the most frequent mutations of *KRAS* are G12VD (31%), G12V (31%), and G12R (21%). Importantly, multiple concurrent *KRAS* mutations were detected in approximately 4% of PDACs, and, interestingly, these different *KRAS* mutations could be presented in different cells of the same tumor (19). As a tumor suppressor, *TP53* is the top frequently mutated gene in multiple tumors. Based on the results of the MSK-IMPACT study and the GENIE project, *TP53* mutations have been identified in approximately 70% of PDACs (20). The missense mutations of *TP53* occurred at approximately 190 codons, and the “hotspot” mutation codons included 175, 245, 248, 249, 273, and 282 whose mutation leads to abnormal conformational changes in the DNA-binding surface of TP53 (21). The mismatch repair–deficient cancers usually generate a large amount of neoantigens that are beneficial for their sensitivity to immune checkpoint blockage (22). It was reported that those pancreatic patients with longer survival time usually have stronger T-cell activity and less immunogenic mutations (neoantigens), which implied that the quality of neoantigens is important biomarker for tumor immunotherapy (18). Moreover, in addition to the location proximity of cytotoxic T cells to cancer cells, the neoantigen number in combination with the CD8^+^ T-cell infiltration status is correlated with the survival of patients with PDAC (23, 24). It was reported that the mis-splicing of exons and errors in microsatellite (MS) transcription could generate highly immunogenic frameshift (FS) neoantigens whose sequence could be predicted and used to build the peptide array that covers all possible FS neoantigens (25).

**Table 1:** The list of mutated genes and the related pathways in pancreatic cancer (PC).

Pathway	Mutated Gene	Global Frequency (%)
KARS	**KRAS**, MAPK4	92
Cell Cycle	**TP53**, CDKN2A	78
TGF-beta Signaling	SMAD3, TGFBR1, ACVR1B, **SMAD4**, TGFBR2, ACVR2A	47
DNA Repair	BRCA1, BRCA2, PALB2, ATM, ATF2	17
Chromatin	MLL2, MLL3, **KDM6A**, SETD2	26
SWI/SNF	ARID1A, ASD1B. PBRM1, SMARCA4	20
Notch Signaling	JAG1, BCORL1, NF2, FBXW7	10
WNT Signaling	RNF43, MARK2, TLE4	5
RNA Processing	RBM10, SF3B1, U2AF1	15
ROBO SLIT Pathway	ROBO1, ROBO2, SLIT2, MYCBP2	5

The table is modified from the paper (18). The top frequent drivers of pancreatic tumorigenesis are indicated in bold.

Given that PDACs are highly associated with several mutations of genes such as *KRAS, TP53*, and *CDKN2A*, the development of neoantigen-based immunotherapy might be a promising therapeutic strategy for the treatment of PDAC. There are two kinds of neoantigens: shared neoantigens and personalized neoantigens (26, 27). Shared neoantigens are common antigens that are present in different cancer patients. Wenyi Zhao et al. found that 10 neoantigens were shared by approximately 50% of pancreatic patients that can be the potential targets for off-the-shelf immunotherapy (27). Personalized neoantigens are the unique mutated antigens from other most frequently used neoantigens, and the targeting of personalized neoantigens is specific personalized therapy (28). More importantly, the hallmarks of carcinogenesis are the accumulation of genetic and epigenetic mutations that are commonly classified as a ‘driver’ or ‘passenger’ mutation according to their roles in promoting cell proliferation and invasion (29). Driver mutations have the capability to drive the cell lineage to cancer, but passenger mutations do not exhibit the proliferation-promoting benefit to cell lineage (30). Moreover, due to the large fraction of passenger mutations compared with driver mutations, the passenger genes contribute to the majority of experimentally confirmed neoantigens that showed high immunogenicity (31). More notably, PCs have a limited number of neoantigen expressions due to their markedly low mutational burden, which is highly associated with the efficiency of immunotherapy (32). In line with this finding, the KPC pancreatic mice models showed that low mutation burden is highly associated with deficient immunoediting, but the ectopic expression of a neoantigen Ovalbumin (OVA) could rescue the tumor elimination ability (33). Therefore, these findings indicated that mutation burden can be considered a potential predictive biomarker of clinical response to checkpoint inhibition therapy (34).

A comprehensive analysis was performed on the genomic profiles of 221 PDAC cases, and the results indicated that the targetable neoantigens were expressed in almost all PDAC samples. Importantly, the top promising targetable neoantigens are KRAS codon 12 mutations (35). It was reported that T-cell receptors (TCRs) that are reactive to KRAS G12V and G12D neoantigens could be isolated for the immunized HLA-A*11:01 transgenic mice. Furthermore, these TCR-transduced peripheral blood lymphocytes (PBLs) could recognize plenty of HLA-A*11:01(+) tumor cell lines expressing the KRAS G12V and G12D mutations (36). Moreover, the genetically engineered T cells ectopically expressing two allogeneic HLA-C*08:02-restricted TCRs targeting KRAS G12D neoantigens were injected into the patient with metastatic PC and regressed the visceral metastases of the patient (37).

## Neoantigen-based vaccines in pancreatic cancer

In recent years, immunotherapeutic strategies including various immune-checkpoint blockage, chimeric antigen receptor T-cell therapies are well studied in the treatment of multiple cancer types including PC. Importantly, as a novel cancer immunotherapy, the development and application of the personalized vaccines based on tumor-specific neoantigens attracted increasing attention in treating diverse cancers by enhancing the endogenous repertoire of tumor-specific T cells (38). Given that the neoantigens are expressed only on the tumor cells but not normal cells and they are unique epitopes of somatic mutations, the neoantigen-based vaccines could avoid the “off-target” damage to normal cells and prevent the T-cell central tolerance caused by self-epitopes and subsequently induce the tumor-specific T-cell response (38). Early studies showed that the neoantigen-based personalized cancer vaccines proved promising outcomes in prolonging the overall survival (OS) of cancer patients. The advances of next-generation sequencing and the bioinformatics for the prediction of major histocompatibility complex class I (MHC I)–binding epitopes promote the identification of tumor-specific mutations and the generation of personalized therapeutic cancer vaccines based on neoantigens.

Notably, the feasibility, safety, and immunogenicity of neoantigen-based personalized cancer vaccines were fully measured in patients with melanoma and glioblastoma (39, 40). The effect of iNeo-Vac-P01, a personalized neoantigen-based peptide vaccine, was examined in the treatment of PC, and the iNeo-Vac-P01 could enhance the clinical efficacy of PC (41). Recently, Li L et al. combined next-generation sequencing, novel predictive modeling techniques, and computational algorithms based on bioinformatics to build and optimize a DNA vaccine platform to target multiple neoantigens in a metastatic pancreatic neuroendocrine tumor (42). In their study, the positive effect of the optimized polyepitope neoantigen DNA vaccine on the induction of antitumor immune responses and neoantigen-specific TCRs were confirmed in preclinical and clinical trials. Moreover, their findings also suggested that the neoantigen DNA vaccine can target multiple neoantigens at the same time and the longer epitope fragments could markedly extend the immune responses. Importantly, they found that the addition of a neoantigen-tagging mutant marker on the end of the epitope could dramatically enhance the immune responses (42). A neoantigen-targeted vaccine was generated using the synthesized 20-mer peptides according to the mutation of 12 genes: Myo1g, Ace, Glb1L12, Map2k5, Rasa3, Clcn7, Notch2, Bsg, Pnpla7, Ppp2r3a, Tg, and Ttn (43). The effect of the triple immunotherapeutic strategy, the combined administration of neoantigen-targeted vaccine PancVAX, anti-PD-1, and agonist OX40 antibodies, was investigated on the treatment of xenograft mice bearing pancreatic adenocarcinoma (Panc02) cells (43). The results indicated that PancVAX led to the transient regression of the tumor by inducing markedly the tumor infiltration of neoepitope-specific T cells. The addition of anti-PD-1 and agonist OX40 antibodies decreased the exhausted T-cell number, induced a durable tumor regression, and prolonged the survival time (43). Furthermore, TG01, the first injectable antigen-specific tumor immunotherapy targeting KRAS mutations, was designed by including seven synthetic RAS peptides that covered seven KRAS common mutations that occurred in codon 12 and 13 (44). The design aimed to activate both MHC-I CD8+ and MHC-II CD4 T-cell functions because the activation of CD4+ cells plays important roles in promoting the DC- meditated cross-presentation of neoantigens and TAAs on the tumor surface and further enhances the antitumor effect of CD8+ T cells (44, 45). Moreover, the safety, immunological responses, and clinical effect for TG01 in combination with recombinant human granulocyte macrophage–colony-stimulating factor and gemcitabine (GEM) chemotherapy were firstly examined on the resected pancreatic adenocarcinoma by Daniel H. Palmer et al., and the result indicated that the vaccination strategy can be well tolerated and trigger remarkable immune responses (45).

Currently, different personalized neoantigen-based cancer vaccines for the treatment of patients with PC are in the clinical trials (**Table 2**). With the advanced development of tumor mutation identification tools, neoantigen-prediction algorithms, vaccine delivery platforms, novel immunogenomic tools, and other bioinformatics technologies, the more effective, long-lasting personalized neoantigen-based immunotherapeutic strategies, especially the neoantigen-based vaccines, will be explored and benefit the patients with PC.

**Table 2:** Clinical trials of neoantigen-based therapies on PC.

NCT Number	Intervention/ treatment	Phase	Start date	Completion date	Enrollment	Status	Sponsor
NCT03645148	iNeo-Vac-P01 + GM-CSF	I	10/24/2017	04/01/2021	7	completed	Zhejiang Provincial People’s Hospital
NCT05111353	Neoantigen peptide vaccine: Poly-ICLC	I	10/10/2022	12/31/2027	30	Recruiting	Washington University School of Medicine
NCT03122106	Personalized neoantigen DNA vaccine	I	01/05/2018	08/13/2022	15	Terminated	Washington University School of Medicine
NCT03956056	Neoantigen peptide vaccine: Poly-ICLC	I	02/13/2020	06/21/2023	12	Active, not recruiting	Washington University School of Medicine
NCT04810910	iNeo-Vac-P01 + GM-CSF	I	03/30/2021	03/30/2025	20	Recruiting	Zhejiang Provincial People’s Hospital
NCT03558945	Personalized neoantigen vaccine	I	04/02/2018	04/30/2023	60	Recruiting	Changhai Hospital
NCT04799431	Neoantigen vaccine with Poly-ICLC adjuvant: retifanlimab	I	01/01/2023	01/01/2028	12(estimated)	Not yet recruiting	Sidney Kimmel Comprehensive Cancer Center at Johns Hopkins
NCT04161755	Personalized tumor vaccines (PCVs) + PD-L1 blocker: atezolizumab + mFOLFIRINOX	I	12/13/2019	11/01/2023	29	Active, not recruiting	Memorial Sloan Kettering Cancer Center
NCT03953235	GRT-C903/GRT-R904 + nivoluma/ipilimumab	I/II	07/18/2019	12/00/2023	144	Recruiting	Gritstone bio, Inc.
NCT03871790	Peptide-based immunization	N/A	04/01/2019	11/01/2021	100(estimated)	N/A	CENTOGENE GmbH Rostock
NCT03662815	iNeo-Vac-P01 + GM-CSF	I	02/07/2018	12/30/2022	30(estimated)	Active, not recruiting	Sir Run Run Shaw Hospital
NCT05013216	KRAS peptide vaccine Hiltonol^®^ (Poly-ICLC)	I	04/11/2022	05/01/2026	25(estimated)	Recruiting	Sidney Kimmel Comprehensive Cancer Center at Johns Hopkins
NCT03468244	Personalized mRNA tumor vaccine	N/A	05/01/2018	12/31/2020	24(estimated)	N/A	Changhai Hospital
NCT05292859	Neoantigen-specific TCR-T-cell drug product	N/A	09/00/2022	06/00/2039	180(estimated)	Not yet recruiting	Alaunos Therapeutics
NCT04117087	KRAS peptide vaccine, nivolumab, ipilimumab	I	05/29/2020	06/01/2024	30(estimated)	Recruiting	Sidney Kimmel Comprehensive Cancer Center at Johns Hopkins
NCT05194735	Neoantigen-specific TCR-T-cell drug product + aldesleukin (IL-2)	I/II	04/04/2022	03/00/2029	180(estimated)	Recruiting	Alaunos Therapeutics
NCT02600949	Synthetic tumor-associated peptide vaccine: imiquimod	I	05/11/2016	05/31/2025	150(estimated)	Recruiting	M.D. Anderson Cancer Center

All clinical trial data were collected from ClinicalTrials.gov by the keywords neoantigen and pancreatic cancer (https://clinicaltrials.gov/ct2).

## Dendritic cell vaccines of pancreatic cancer

DC–mediated antigen presentation plays essential roles in modulating immune responses. The dysregulation of antigen processing and presentation is a critical mechanism facilitating tumor escape from the immune surveillance by the immune system. However, it was reported that DCs and other antigen-processing and -presenting molecules such as human leukocyte antigen (HLA) class I and transporter for antigen presentation (TAP) were downregulated in PCs (46). Moreover, accumulating evidence indicated that tumor antigen–based DC vaccines exhibited effectiveness to induce the T-cell-mediated adaptive cytolytic immune responses in PC (46).The development of DCs vaccines aimed to connect the DCs with TAA to present the antigen and subsequently activate cytotoxic T cells. Currently, various types of DC-based vaccines were developed and in the clinical trial stage (47).

In a phase I study, the effect of allogeneic tumor lysate–loaded autologous monocyte-derived DCs was evaluated in the treatment of resected PDAC and the vaccination treatment indicated a feasible and safe immune reactivity induction capability (48).The prophylactic DC vaccination strategy used DC vaccines generated by *ex vivo* differentiation, and the maturation of bone marrow–derived precursors was investigated in PDAC tumor mice models and exhibited a significant effect in inhibiting recurrent tumor growth and extending the survival time (49). It was well known that in PCs, the telomerase [human telomerase reverse transcriptase (hTERT)] is a promising target antigen and it mainly expresses in cancer stem cells that are difficult to eliminate by common therapeutic strategies (50). PC patients who received the vaccination of DCs transfected with hTERT full-length mRNA exhibited an induction of hTERT-specific immune responses but have not experienced serious adverse issues. This study therefore provided positive evidence for the generation of DC vaccines loaded with mRNA for a specific antigen with clinical relevance by inducing the antigen-specific immune responses (51). However, although DCs are well known for their antigen presentation function in the immune system and could induce the TCR specific to tumor antigens, the immunotherapeutic efficacy of DCs in combating PCs is still limited. Therefore, the combination immunotherapy of DC vaccines with other therapies to improve the efficacy of DC vaccination is a promising research topic. In line with this notion, the efficacy of peptide-pulsed DC vaccines in combination with the Toll-like receptor (TLR)-3 agonist poly-ICLC was tested in the murine orthotopic Panc02 cell models. The combination vaccination method showed a significant antimetastatic effect *via* CD8+ T-cell activation (52). The DC vaccines could also dramatically induce the cytotoxic T-lymphocyte (CTL) responses and block the migration of PC (53). Given that numerous antitumor strategies including checkpoint inhibitor treatments are less sensitive to PDAC, the combined therapeutic method by inducing tumor-specific T cells *via* DC vaccination and remodeling the desmoplasia of the tumor microenvironment (TME) *via* CD40-agonistic antibody administration could reduce the tolerance to PDAC. Therefore, the effect of mesothelioma-lysate loaded DCs coupled with FGK45 (CD40 agonist) was tested in immune-competent PDAC mice models and the novel approach induced a significant change in the tumor transcriptome including the inhibitory markers on CD8 +T cells and dramatically enhanced patient survival (54). Notably, the survival of patients with advanced pancreatic carcinoma who received the DC-based immunotherapy combined with GEM and/or S-1 was dramatically prolonged by the administration of lymphokine-activated killer (LAK) cell therapy. However, immunotherapy alone could increase the number of cancer antigen–specific cytotoxic T cells and reduce the regulatory T cells (55). The antitumor effect of DCs loaded with alpha-galactosylceramide (alpha-GalCer) was evaluated in PC C57BL/6 mice models, and tumor growth was inhibited, which might be correlated with the increased number of IFNγ-producing NKT cells (56).

Currently, most of the efforts of DC-based vaccination were focused on MUC1, WT1, and KRAS antigens. The TAA MUC1, a glycoprotein, is ubiquitously expressed in PC cells. The effect of MUC1-DCs vaccine that was generated by transfecting the liposomal MUC1 cDNA into DCs was investigated in PC patients, and the result indicated that MUC1-DC vaccination was well tolerated and enhanced the CTL response (57). However, MUC1 peptides-loaded/pulsed DC vaccines were well tolerated in treating PCs in two separate studies but the clinical benefit is controversial, which might be caused by the different patients (58, 59). Importantly, the efficacy of triple therapy for MUC1-mRNA-transfected DCs in combination with MUC1-CTLs and GEM was examined in PCs and the addition of MUC1-DCs and MUC1-CTLs dramatically prolonged the survival time (60). The Wilms tumor gene (WT1) is overexpressed in many PCs, and WT1 peptide–pulsed DC vaccines were reported to dramatically prolong the median OS of PC patients in combination with standard chemotherapy (61).

## Neoantigen-based dendritic cell vaccines in pancreatic cancer

Generally, neoantigens belong to antigens, but they are specific novel antigens to each patient’s cancer and produced by random mutations in the cancer genome. Therefore, DCs still own the capability to present the neoantigens to T cells and subsequently induce a specific immune response to each patient with related mutations. The high level of neoantigen expression is positively correlated with pathogenic TCR and PDAC progression. The dysregulation of conventional DCs (cDCs) is highly correlated with abnormal immune surveillance and hampered the response of early T_H_1 and CTL to the neoantigens of PDAC (62). Moreover, the function and amount of cDCs in PDAC could be considered as a biomarker for the adaptive immune responses to tumor neoantigens in PDAC (63). Therefore, DCs may play essential roles in neoantigen presentation and inducing the neoantigen-specific TCR because the neoantigen-loaded DC vaccines can directly present neoantigens to T cells.

Due to the unclear mutational load of specific cancers, the large amount of DC vaccines was designed to induce the immune response by targeting predetermined and universal antigens. However, the neoantigen-based DC vaccine is considered a personalized DC vaccine because the patient-specific neoantigens could be identified through novel genomic sequencing technologies and bioinformatics (64). Given that PC is characteristic for lower tumor burden and a limited number of neoantigen and DCs, the effect of neoantigen-pulsed DC vaccines was firstly examined in other cancers including melanoma and lung cancer. The induction of the neoantigen-specific TCR repertoire of neoantigen-pulsed DC vaccination was firstly investigated in advanced melanoma (65). Moreover, the feasibility, safety, immunogenicity, and efficacy of a peptide vaccine targeting 20 predicted neoantigens were further investigated in melanoma patients, which provided a newly possible rationale to optimize the neoantigen-based vaccine and the development of novel therapeutic strategy in combination with commonly used immunotherapies (39). Notably, the effect of neoantigen-pulsed DC vaccines on the patients with advanced NSCLC was evaluated in a trial in 2021 (11). They generated the personalized DC vaccines based on the 13-30 peptide of the neoantigens identified in the tumor tissues of 12 patients. Upon the personalized neoantigen-pulsed DC vaccine treatment, 25% of patients responded to the vaccination and 75% of patients showed a disease inhibition, which indicated a favorable therapeutic outcome for the vaccination strategy (11). Furthermore, Changbo Sun et al. found and designed a neoantigen short peptide L82 based on the result from the whole-exome and RNA sequencing on the LLC1 cell line. This candidate neoantigen short peptide L82 can both trigger CD8^+^ T-cell responses and suppress the LLC1 growth *in vivo*. THE L82-pulsed DC vaccination in combination with anti-CD38 antibody treatment effectively inhibited the tumor growth by reducing the tumor- infiltrated regulatory T cells (66). Furthermore, the effect of the combination of DC-loaded with MART-1 peptide vaccine with tremelimumab and the anti-CTLA-4 antibody was tested in melanoma, and the treatment strategy achieved a stronger and durable tumor response than each treatment alone (67). Moreover, the patients with metastatic gastric cancer have received the administration of neoantigen-pulsed DC (Neo-MoDC) vaccines combined with immune checkpoint inhibition (ICI). Although the Neo-MoDC vaccine alone could trigger neoantigen-specific CD4^+^ and CD8^+^ TCRs, the combination therapy induced a higher immune response and significant elimination of tumors (68). Notably, a phase Ib trial (CHUV-DO-0017_PC-PEPDC_2017) was also conducted on a DC vaccine pulsed with personalized neoantigen peptides (PEP-DC) coupled with the treatment of chemotherapy and the anti-PD-1 antibody. The study comprehensively evaluated the feasibility, safety, immunogenicity, and efficacy of the combination therapy of the DC vaccine in PCs (69). Therefore, these findings suggested that the neoantigen-based DC vaccine combined with other therapeutic strategies enhanced the effects of cancer treatment by inducing highly patient-specific immune responses and provided novel therapeutic opportunities for cancer treatment.

In a phase I pilot study, the feasibility and efficacy of a DC vaccine pulsed with the Wilms tumor gene-1 (WT1) peptide in combination with GEM were evaluated in the treatment of advanced PC as a first line of treatment. The WT1 peptide–pulsed DCGEM is feasible and effective in triggering the antitumor TCRs but showed less effect in treating the PC with live metastasis and elevated levels of inflammatory markers (70). Furthermore, a phase Ib trial (CHUV-DO-0017_PC-PEPDC_2017) was conducted to test the safety, immunogenicity, feasibility, and efficacy of the DC vaccine pulsed with personalized neoantigen peptides (PEP-DC) in PDAC and the efficacy of combination with aspirin, nivolumab, and adjuvant chemotherapy was further evaluated (69). NeoDisc, a novel proteogenomics antigen discovery pipeline, was used to find and optimize the candidate neoantigen in PDAC, and, furthermore, the long peptides of relative neoantigens were designed. Due to the possibility of low immunogenic capability, the p53 (TP53), mucin-1 (MUC1), prolyl endopeptidase FAP (FAP), TAA mesothelin (MSLN), outer dense fiber protein 2 (ODF2), coiled-coil domain-containing protein 110 (CCDC110), and the testis-specific protein bromodomain testis-specific protein (BRDT) were excluded in the design of the DC vaccine (69). Shikhar Mehrotra et al. generated a DC vaccine pulsed with three specific A2-restricted peptides: 1) hTERT (TERT572Y), 2) carcinoembryonic antigen (CEA; Cap1-6D), and 3) survivin (SRV.A2). The neoantigen-pulsed DC vaccine in combination with the Toll-like receptor (TLR)-3 agonist poly-ICLC was used to treat the metastatic or locally advanced unresectable PC (52). The results of the study indicated that the combination therapeutic strategy is safe and could effectively induce the tumor-specific TCR (52). Furthermore, the MUC1 peptide-pulsed DC vaccines were used to treat the advanced PC patients and the results indicated that the vaccination is safe and effective to trigger the immunological response to the tumor antigen MUC1 (58, 59).

Importantly, the effects of the mDC3/8-KRAS vaccine that included a DC vaccine loaded with KRAS mutation peptides on the resected PDAC patients is evaluated in the ongoing phase I study (NCT03592888). For each vaccine dose in the trial, all subjects will receive autologous DCs pulsed with mutant KRAS peptides corresponding to the subject’s specific tumor mutation and HLA type. In addition, the inclusion criteria included pathologically confirmed KRAS(G12D-), KRAS(G12V-), KRAS(G12R-), or KRAS(G12C-mutated) PDAC who are at a high risk of relapse and have no evidence of disease (NCT03592888). A terminated phase II trial on a DC vaccine against defined neoantigens expressed by autologous cancers had been performed in patients with epithelial cancers including PDAC, but only one patient was enrolled (NCT03300843).

Therefore, given that PDAC is one of the poorly immune-responsive cancers and some currently used immunotherapies are also less effective in treating PDAC, it is urgent to develop novel therapeutic strategies including alone administration or the combinations of chemotherapy, radiation, vaccines, and ICIs and the development of personalized neoantigen-pulsed DC vaccines to fully induce antitumor TCRs.

## Conclusion and outlook

Recently, immunotherapies have proven their effectiveness in multiple types of cancers but the efficacy is limited in the treatment of PC partially due to the immune-tolerant state, lower mutational burden, and decreased amount of DC. The high tumor specificity and immunogenicity make neoantigens an exciting and promising target of tumor immunotherapy. Neoantigens, the novel and specific antigens, could also be presented by DCs. Neoantigen-based DC vaccines illustrated promising effects in multiple cancers, but few are investigated in PCs, which is possibly due to low mutation burden and limited neoantigens. Given that the feasibility, safety, immunogenicity, and efficacy of neoantigen-based DC vaccines were evaluated in various cancers, it is an urgent need to put more effort on the development of suitable neoantigen-based DC vaccines for PC and evaluate their efficacy in clinical trials, which will provide precise treatment for more PC patients. However, high costs, longer time for manufacturing, difficulty in large-scale DC cell maturation, the low efficiency of DC migration, the lack of a better method for accurate identification of immunogenic neoantigens, and a less optimized vaccine delivery platform limited the development of personalized immunotherapy, which warrants further in-depth investigation in the future (38). Fortunately, with the advances and optimization in whole exome sequencing and neoantigen-prediction algorithms, the in-depth understanding of the molecular mechanisms underlying the immune-tolerant state of pancreatic cancer, the development of neoantigen-based DC vaccines will be significantly improved. The combined immunotherapy approach using neoantigen-based DC vaccines, chemotherapy, and ICIs showed exciting therapeutic benefits to various cancer patients. Therefore, the novel therapeutic strategies including combination immunotherapy for PC should also be explored and optimized to benefit more PC patients.

## Author contributions

XinZ wrote the manuscript with partial help from ZX. XD, XiaZ, and XW edited and revised the manuscript. All authors approved the final manuscript.
